# Visualization and quantification of facial muscles with 9.4T MRI-DTI in common marmosets (*Callithrix jacchus*)

**DOI:** 10.1098/rsos.251134

**Published:** 2025-11-12

**Authors:** Kanako Muta, Catia Correia-Caeiro, Junichi Hata, Takako Miyabe-Nishiwaki, Sho Kurihara, Yuri Takae, Hirotaka James Okano, Hideyuki Okano, Anne Burrows

**Affiliations:** ^1^ Graduate School of Human Health Science, Tokyo Metropolitan University, Tokyo, Japan; ^2^ The University of Tokyo Graduate School of Agricultural and Life Sciences/Faculty of Agriculture, Tokyo, Japan; ^3^ Center for the Evolutionary Origins of Human Behavior, Kyoto University, Inuyama, Aichi, Japan; ^4^ Human Biology & Primate Cognition, Leipzig University, Leipzig, Sachsen, Germany; ^5^ Connectome Analysis Unit, Center for Brain Science, RIKEN, Saitama, Japan; ^6^ Division of Regenerative Medicine, The Jikei University School of Medicine, Japan; ^7^ Department of Physiology, Keio University School of Medicine, Tokyo, Japan; ^8^ Division of Regenerative Medicine, The Jikei University School of Medicine, Tokyo, Japan; ^9^ Department of Otorhinolaryngology, The Jikei University School of Medicine, Tokyo, Japan; ^10^ Department of Physical Therapy, Duquesne University, Pittsburgh, PA, USA; ^11^ Department of Anthropology, University of Pittsburgh, Pittsburgh, PA, USA

**Keywords:** anatomy, facial muscles, diffusion tensor imaging, common marmoset, muscle anatomy, diffusion MRI

## Abstract

The traditional dissections of the specialized mimetic muscles that produce facial expressions have revealed important insights into the evolution of muscles and their function in human and other mammals’ social interactions. However, in small species, the task of manually visualizing and analysing musculature is challenging; even in larger species and muscles, digital anatomy methods, such as DiceCT and MRI-DTI, have been growing in popularity recently, providing detailed new insights into the structure and morphology of muscles. The current work presents for the first time a 9.4T MRI-DTI visualization and analysis of the complete facial musculature used for facial expression in a primate species, in this case a very small species hard to analyse with traditional dissection, the common marmoset. In addition, a quantification and comparison of overall, individual and bilateral muscles was performed in the light of functional anatomy, followed by a critical analysis of this novel method for the study of facial anatomy. Twenty-two muscles were tracked, described, quantified and compared in three individuals. This work demonstrates the feasibility of MRI-DTI as a relatively novel method to digitally visualize the structure of facial muscles in a small species. In addition, this method is able to quantify varied muscle parameters for comparisons between individuals, muscles and hemifaces.

## 1. Introduction

Facial muscles have been extensively studied due to their important role in producing displays involved in communication and emotion, particularly in humans and other primates. When compared to other mammalian species, primates are considered to have the most complex visual communicative repertoire [1], from which facial expressions play a central role. Investigating the morphology, anatomy and physiology of specialized mimetic muscles responsible for facial expressions has provided valuable insights into muscle evolution and their function in social interactions among humans and other primates. For example, researchers discovered that the human facial muscles are adapted for slower movements than chimpanzee and macaque muscles, which allowed the development of intricate and highly controlled mouth movements for speech [2].

However, the facial musculature is a very thin layer of several superimposed individual and small muscles tightly arranged between the dermis of the skin and the bones of the skull. Dissecting these muscles requires high expertise to peel the skin and carefully separate each muscle individually, but due to the size and tight arrangement, this is not always a successful task. In fact, to the untrained eye of a non-expert anatomist in facial musculature, facial muscles look almost like a continuous single layer of flesh, hiding its complexity and important function (e.g. [3]). The examination of facial muscles in small primate species is thus an even more challenging task. Therefore, even though small primates such as the common marmoset (*Callithrix jacchus*) are very abundant in captivity and have been extensively studied both in the field [4,5] and in the laboratory [6–8], only one modern dissection of one individual of this species’ facial muscles has been published so far [3].

Common marmosets are small arboreal New World primates that live in highly social and cooperative groups [5,9], with extensive visual behaviour repertoires [5,10]. In particular, and contrary to what was previously thought [3,11], common marmosets have a high number of facial movements [12] and a complex range of facial displays used in varied contexts [13]. Due to this species' behavioural, anatomical and morphological characteristics, there is also great interest in comparative studies between common marmosets and humans (e.g. for understanding higher brain mechanisms that underlie emotion and communication common in primates [14]). Hence, the study of their facial muscles is of scientific interest. Furthermore, due to the small size (e.g. skull size of humans: 17.41 × 14.03 cm [15] versus common marmosets: 5.2 × 3.4 cm [16]), and the difficulty to perform dissections and visualize the facial muscles, this is a good model species to develop other methods of visualizing facial muscles in common marmosets. Developing tools to aid in this task with such a small species will also help further research in the evolution of human and other primates’ social behaviour.

To avoid the difficult task of separating the facial skin from the facial muscles, the technique of Reverse Facial Mask dissection has been developed [17] and has been reported as more effective to investigate the morphology and anatomy of the thin and intricate mimetic musculature in primates [17–19]. In this technique, the facial mask is separated from the skull and inverted, so all muscles are conserved and easily visualized. However, the Reverse Facial Mask technique still requires partially destroying the tissues to separate and identify the individual muscles, rendering the sample unusable for repeat dissections or other purposes. In addition, it is time-consuming and still requires high anatomical expertise [17].

Although dissections have traditionally been a strictly manual process, researchers have been exploring the use of existing imaging technology and equipment to complement, and in the future, potentially replace manual dissections. One of these digital techniques is the Diffusible Iodine-Based Contrast-Enhanced Computed Tomography imaging technique (DiceCT), which has been adapted from regular X-ray CTs used for mineralized tissues (e.g. bones) by adding a soft tissue staining iodine agent to allow radiopacity in soft tissues [20]. Initially used for three-dimensional imaging of the soft tissue of embryos [21], its use rapidly expanded to target diverse soft tissues in a wide range of species, while the technique itself was being refined (reviewed in [20]). In particular, DiceCT has been used for examining head and mastication musculature in different species (e.g. squirrel, guinea pig and rat [22]; bat [23]; aye-aye and mongoose lemur [24]; common marmoset [25]; crab-eating macaque [26]; mouse and squirrel [27]; pig [28] and penguin [29]). However, to the best of our knowledge, this technique has only been applied to the full mimetic musculature of a primate species by Dickinson *et al*. [30]. Unlike the Reverse Dissection, DiceCT conserves the musculature form and allows for more precise quantitative measurement of the muscle’s fascicles (i.e. muscle dimension and volume) and its relative function (including force production ability). Despite the DiceCT requirement for muscle staining, it is a reversible and non-destructive technique [20], which has allowed identification of muscles previously missed in reverse dissections [17]. More importantly, being an imaging technique, it allows digital visualization, which means augmentation, three-dimensional rotation, coloration for easy labelling, ease of sharing and repeated analysis of the same specimen become effortlessly attainable. However, this technique still requires a manual step of prolonged muscle staining (approx. 2 weeks), which presents specimen variability and may be somewhat time-consuming and labour-intensive [17]. The staining time can be variable depending on several factors, such as size, depth of tissue to stain and concentration of staining solution [20]. In addition, both Reverse Dissection and DiceCT can only be done post-mortem, so either researchers need to wait for opportunistic sampling of natural deaths, euthanasia for other purposes (e.g. other research projects, terminal diseases) that do not damage the head and facial musculature, or individuals need to be euthanized, which is less than ideal and, for the latter case, even unethical.

As a complement to macro-anatomical techniques such as the ones mentioned above, histological techniques can provide further insight into the microanatomy of muscle tissue and, in particular, towards the understanding of the architecture of facial muscles. Studies have found significant micro-anatomical differences between ape species relevant for evolutionary comparisons, which the macro-anatomy alone could not reveal [31–33]. Histological and digital techniques combined can also be used to quantify facial muscle fibres, for example, to measure the volume of tissue damage [34], to compare proportions of slow- and fast-twitch fibres in neighbouring muscles [35] and to understand processes of ageing in facial tissues [36]. However, histological techniques require, more than the macro-anatomical techniques, destruction of the tissues through sectioning and staining and are usually applied to very small portions of a muscle.

A promising imaging method combined with sophisticated analytical techniques has been developed in the last decade: the Diffusion Tensor Imaging (DTI) analysis. This method makes use of a parameter called Diffusion Weighted Imaging (DWI), which can image and measure the diffusibility of water molecules within tissues. Based on this DWI parameter, the DTI analysis can image diffusion anisotropy of the water molecules and estimate the direction, number, or length of fibrous structures, such as nerves or muscles. The DTI analysis of the Magnetic Resonance Imaging (DTI-MRI) consists of processing the diffusion-weighted (i.e. with high-contrast resolution) MRI data of water molecules contained within the tissues. In addition to this, Diffusion Tensor Tractography (DTT) can also be specifically used to track fibres within tissues such as muscle or nerve fibres, by calculating a range of parameters, including the magnitude of the diffusion and the direction of water molecules. Colour maps can then be created based on the directionality of the fibres and thus individualizing bundles of fibres in which the direction of the water molecules is the same (see [37] for a review on the DTI and DTT-MRI methods). For facial muscles in particular, DTT is thus an ideal method, since the colour-coding of tightly packed and intertwined muscle bundles according to their direction (and without the need for tissue staining), creates a visually appealing image and greatly facilitates the examination of the different facial muscles. Finally, this technique has the unique benefit of allowing *in vivo* application, in which individuals can be imaged during anaesthesia (e.g. for annual health checks or other necessary procedures). Changes throughout life, such as developmental or clinical changes in musculature, can thus be recorded. For example, DTI/DTT-MRI can detect muscle fatigue [38] and loss of muscle function after tumour resection [39], as well as helping to understand the complex function of the pelvic floor [40] and uterine muscles [41] with clinical importance.

In the present work, we describe the first application of a 9.4T MRI with DTI and DTT analysis to identify, define and visualize facial muscles in the common marmoset. The ultra-high-field 9.4T MRI has high sensitivity and resolution (able to image structures up to 30 µm, [42]) but its use is still not widespread and mostly limited to small animals or body parts (e.g. there are only four large bore 9.4T MRI machines in the world [43]). The obtained images were compared to the results of the Reverse Face Dissection technique [17] used on a common marmoset specimen [3] and were further analysed by a facial muscle anatomy expert (AB) to verify its accuracy and potential for DTI to be used as a methodology for investigating facial muscles in mammals. Given methodological advances, this method has the potential to be developed beyond small-sized animals such as marmosets and applied to larger species for comparative purposes.

## 2. Material and methods

### 2.1. Individuals

The specimens utilized in this study were from three common marmosets (*Callithrix jacchus*) housed at Jikei University and RIKEN. These specimens were used and euthanized for other previous studies by one of the co-authors of the current work [44,45], hence, no individuals were euthanized for the purpose of this study. Post-mortem, the heads were immediately detached from the cervical region and fixed with 4% paraformaldehyde in phosphate-buffered saline for 2 weeks. Please refer to the previous studies for details of euthanasia and further fixation procedures.

The three marmosets (nos. 102, 104 and 106) included in this study were 3, 2 and 2 years old, respectively, and all males. Their body weight when they were euthanized was 129, 262 and 349 g, respectively. Their skull measurements were as follows: no. 102: 35.88 × 20.58 × 15.46 mm, no. 104: 36.65 × 21.09 × 15.12 mm, no. 106: 36.34 × 22.45 × 15.13 mm (long axis × short axis × height).

### 2.2. Image acquisition

The specimens were extracted from formalin, thoroughly dried and placed inside a specially designed container, which was filled with Fluorinert and sealed with a lid, and any residual air pockets were eliminated using a degasser. The sealed container was then introduced into the MRI machine for imaging. The imaging modality employed was DTI using a 9.4T ultra-high field MRI system (Bruker BioSpin, Ettlingen, Germany) with a maximum gradient strength of 660 mT m^−1^ on each axis. An 86 mm volume coil (Bruker BioSpin MRI GmbH, Ettlingen, Germany) was utilized for marmoset specimens. Diffusion-weighted imaging data were acquired using a three-dimensional spin-echo sequence based on a Stejskal–Tanner diffusion preparation. The scanning parameters were as follows: repetition time, 500 ms; echo time, 27.8 ms; flip angle, 90°; field of view, 56.7 mm × 80.5 mm × 26.3 mm; acquisition data matrix, 165 × 230 × 75; reconstructed image resolution, 0.35 mm × 0.35 mm × 0.35 mm; *b* value, 3000 s/mm^2^; motion-probing gradient orientations, 30 axes; motion-probing gradient duration/separation, 6/12 ms; number of repetitions used for signal averaging, 1; and scan time, 77 h.

### 2.3. Image analysis

#### 2.3.1. Pre-processing

Raw MRI data contain many biases and noise, and hence, these data need to be treated before analysis. MRI systems apply a pulsed current to the gradient magnetic field coil during measurement, and residual magnetism is generated as these currents increase or decrease, which can deteriorate MRI sensitivity. In order to improve image uniformity, differences in signal intensity need to be reduced by correcting sensitivity irregularities caused by the residual magnetism. Hence, sensitivity correction was performed using *N4biascorrect* processing [46].

To simplify the analysis and remove the fibre structures of the central nervous system, the brain and spinal cord were masked and removed from the data with BrainSite (https://brainsuite.org [47]). The imaged data after treatment were then segmented and analysed.

### 2.4. MRI image segmentation and fibre tracking

A three-dimensional reconstruction technique to assess fibre tracts using DTI data was used with the open-source software TrackVis, a diffusion toolkit v. 0.6.1 (trackvis.org, Martinos Centre for Biomedical Imaging, Massachusetts General Hospital, Boston, MA). Within TrackVis, grouped muscle fibres are represented in voxels (approx. 50 μm^3^ to ex vivo and approx. 200 μm^3^ to *in vivo* analysis [14]) on images in proportion of muscle fibre number. These voxels can then be tracked using regions of interest (ROIs) defined by the user in infinite three-dimensional spatial plans according to the directionality and length of the voxels. The layer of soft tissue superficial to the bone (henceforth soft tissue), which contains the facial musculature, is clearly visible on the images. For facial muscle analysis, fibres not in this layer of soft tissue are deleted, and only fibres within this layer are tracked (to exclude other fibrous tissues being tracked or tissues that can have water molecules moving, for example: sinuses).

In the current work, T2-weighted (which enhances the signal of the water [48]) and DTI images were merged, and facial muscles were identified as those fibres whose entire length was situated within the layers of soft tissue. For the purposes of the current work, facial muscles were defined as the muscles which sit between the bone/deep fascia and the epidermis to move features of the face. We focused on depicting all the muscles that are more relevant to the production of facial expressions, or more specifically, Action Units (AUs, independent movements) for this species [12]. In addition, we also tracked the temporalis muscle to aid in the spatial localization of the facial muscles.

To select tissue fibres over noisy data, several values of the ‘skip’ parameter were tested. The data were treated with 80%, 90%, 93%, 95% or 98% of skip value to select the most appropriate skip value (see electronic supplementary material, figure S1). Furthermore, the skip value was selected to preserve as much fibre as possible from the muscles of interest, i.e. the skip value was selected so that the fibres situated within the layers of soft tissue were the most likely to remain after treatment, while other fibres were more likely to be deleted or reduced. In the data with a skip value of 0%, there are many noise fibres, making it difficult to recognize and segment muscle fibres, while in the data with a skip value of 98%, muscle fibres that are not considered noise are skipped. The skip value of 93% was therefore selected by KM as it could remove the most noise while preserving the highest number of muscular fibres.

**Figure 1.**
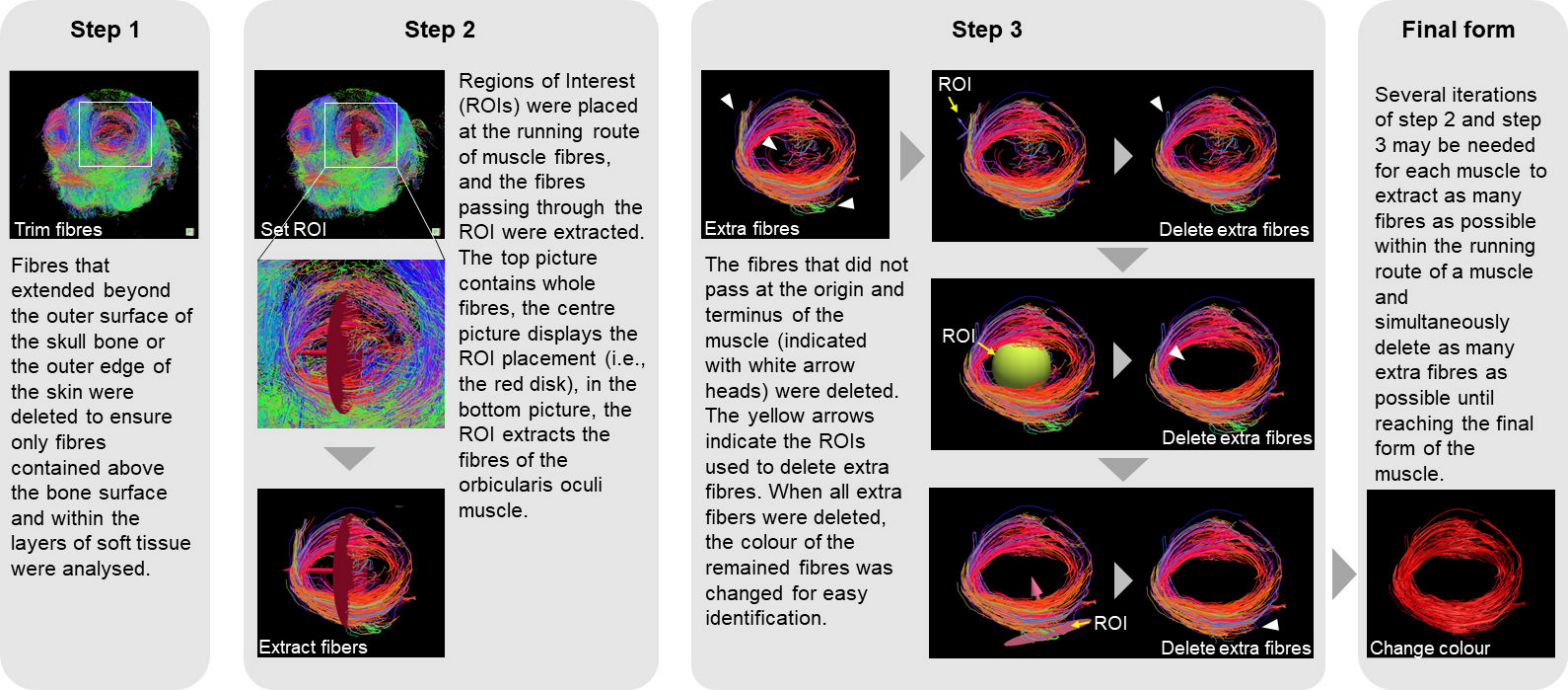
Diagram illustrating an example of the DTI analysis of the orbicularis oculi muscle, with detailed steps, including ROI placement, fibre extraction, extraneous fibre deletion and colour change.

Whenever one end of a fibre was within the layers of soft tissue superficial to the bone, but the other end was not, it was considered to not be part of the facial musculature, and thus it was excluded from analysis. Hence, the image analysis involved the following steps (figure 1):

(1) Removing fibres located within the layers of soft tissue superficial to the bone from the total tracking data.
(2) Placing ROIs along the running route of the muscle fibres, as described in table 1.
(3) Extracting muscle fibres that pass through each ROI. Fibres that did not pass at the origin and insertion points (table 1) were excluded.

**Table 1.** Anatomical description of the head muscles of the common marmoset, including its function, origin and insertion [3,49] used to support the tracking of fibres in the current work. These are the muscles more relevant for the production of AUs in common marmosets [12].

muscle name	abbreviation	function	origin	insertion
Frontalis	F	pulls glabella up	fascia on frontal area	browridge area
Corrugator supercilii*	CS	pulls browridge lateral portions towards the midline	medial end of the superciliary arch	skin of the browridge
Depressor supercilii	DS	pulls glabella down	between OOc and nose	frontalis muscle/browridge area
Procerus	Pro	pulls glabella down	skin of nasal bone	frontalis muscle/browridge area
Orbicularis oculi	OOc	closes eye	circular muscle; attached to skin of eyelid and superciliary region; attached medially to frontal and maxilla bones at the medial palpebral region	
Nasalis	N	opens/closes nostrils	nostrils and along the nose	
Orbicularis oris	OOr	several lips movements	circular muscle; cover upper and lower lip and merge with several lower face muscles	
Zygomatic major	ZMa	retracts lip corners	outer part of zygomatic arch bone	lip corners
Zygomatic minor	ZMi	raises lateral part of lips (not the middle)	medial part of zygomatic arch bone/under OOc	upper lip lateral area
Levator labii superioris alaeque nasi*	LLSAN	wrinkles skin along nose/pulls nostrils up	brow region or just below it	nostrils, side of nose and/or upper lip
Levator labii superioris	LLS	pulls lips up (including medial part)	under the eye/medial OOc	upper lip (sometimes also side of nose)
Superior auriculo labialis	SAL	pulls lips, particularly the lateral portions towards the ears	upper lip, lateral area	anterior to the ear
Depressor anguli oris	DAO	pulls lip corners down	mandible	mouth corner
Depressor labii inferioris	DLI	pulls lower lip down	inferior border of mandible	lower lip
Mentalis	M	pushes chin up	on the mandible, below the canine teeth	skin of mental region (chin area)
Anterior auricularis	AA	pulls ears forward	anterior to the ear (between eye and ear)	pinna
Posterior auricularis	PA	pulls ears backwards	posterior to the head bone, occipital bone	pinna
Superior auricularis	SA	pulls ears upwards	lateral edge of occipitalis muscle fascia	pinna
Depressor helicis	DH	pulls ears downwards	platysma in the neck area	pinna
Platysma	Pla	tightens the neck	around the neck	into several parts of the face and blends with other facial muscles
Temporalis	T	jaw movements (not a mimetic muscle)	temporal bone	mandible
Occipital belly	O	scalp retraction	neck area	fascia on the occipital bone area

Asterisk (*) indicates muscles not previously identified in marmosets, but found in humans and identified in the current work.

This study represents a pioneering endeavour to elucidate the anatomy of marmoset facial muscle fibres in more detail, the specifics of which were previously unknown. Given this absence of information regarding the running of some muscle fibres, inferences were drawn based on previous common marmoset dissections [3,49] to support the identification of the muscle fibres in the current work (table 1). Please note that table 1 lists the muscles described in previous dissections that are more relevant to the production of AUs [12]. These dissections included a small number of individuals (two), so it is possible that due to individual variation, it may differ from our data.

After identification of all facial muscles in all individuals, several of the TrackVis parameters were extracted to quantify the muscle fibres tracked and compare between muscles, individuals and the right and left hemiface (for the muscles that could clearly be separated between hemifaces), namely: track count (i.e. number of tracked artificial fibres), Voxel Count (i.e. number of voxels that fibres passed through), volume (i.e. the volume of a voxel—0.17 × 0.17 × 0.17 = 0.004913 mm^3^—multiplied by the voxel count) and mean length (i.e. The DTI has information on the angle of movement of water molecules for each voxel, and it can be expected that the distance of passage is calculated for each voxel based on the angle information and the size of the voxel; the length of one fibre is the sum of these; the mean length is the mean of all fibres).

## 3. Results

**Figure 2.**
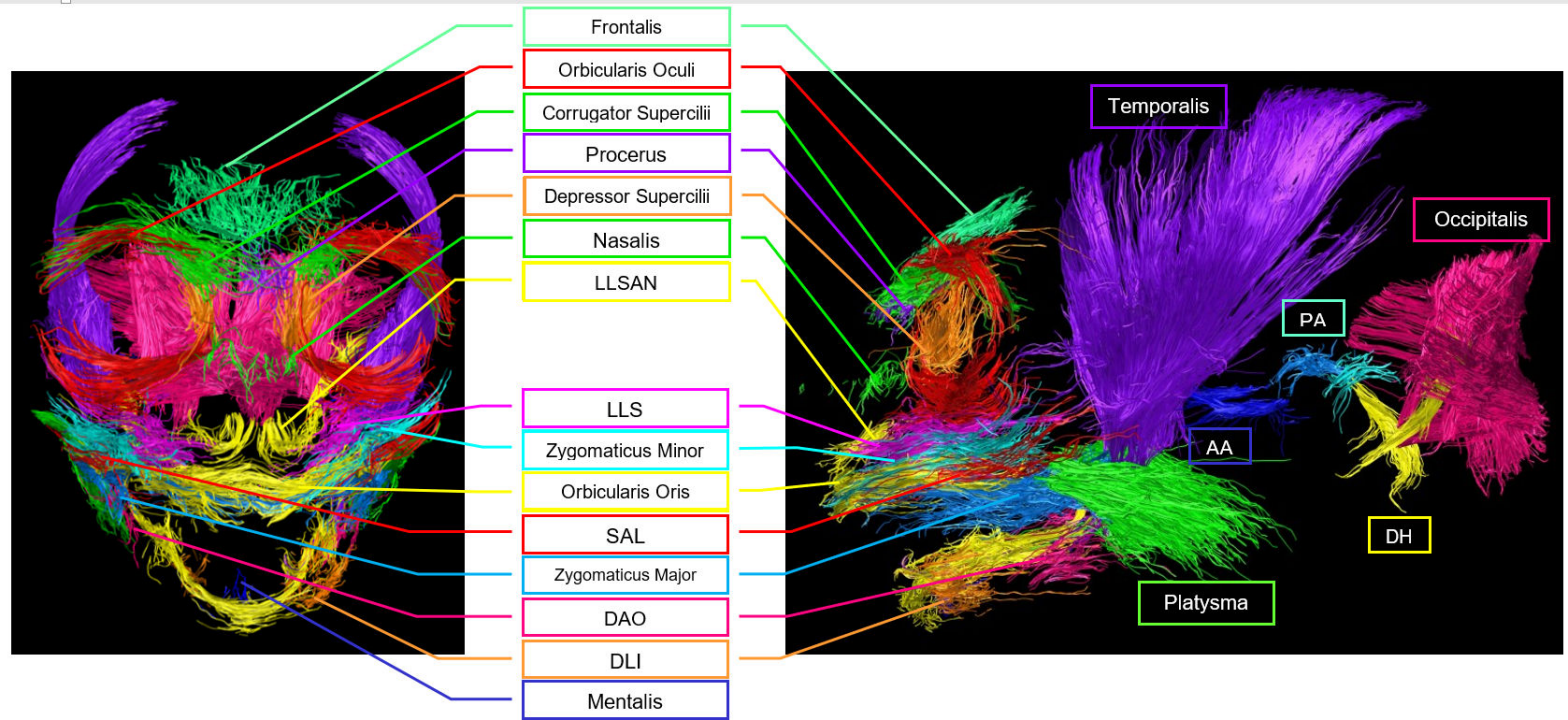
Frontal and lateral view of tracked fibres for facial musculature of individual no. 102. Abbreviations: AA: Anterior Auricularis, DAO: Depressor Anguli Oris, DH: Depressor Helicis, DLI: Depressor Labii Inferioris, DS: Depressor Supercilii, LLS: Levator Labii Superioris, LLSAN: Levator Labii Superioris Alaeque Nasi, PA: Posterior Auricularis, SAL: Superior Auriculolabialis. Green muscle labelled as platysma, but see Limitations section—Identification of muscles, for the masseter as an alternative possibility. Similarly, blue muscle labelled as zygomaticus major, but see the Limitations section for some fibres belonging to masseter being a possibility.

**Figure 3.**
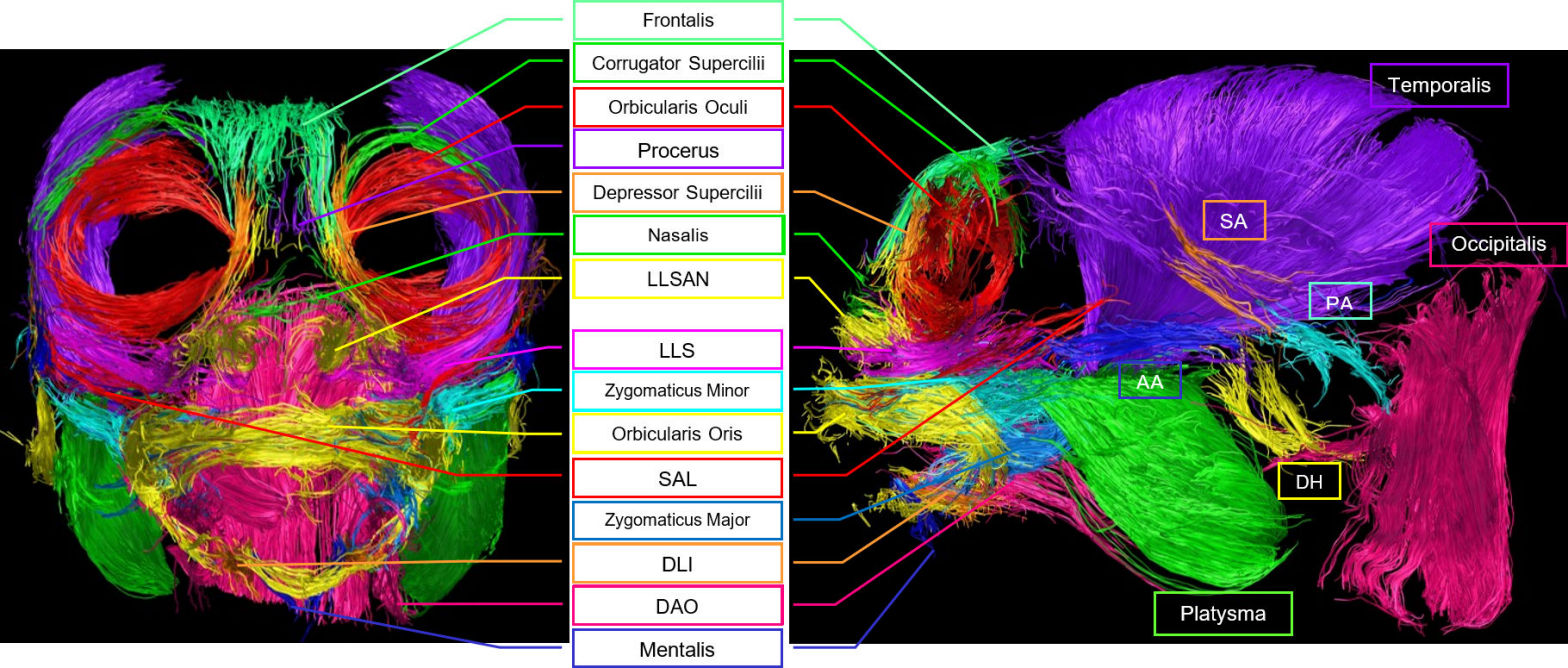
Frontal and lateral view of tracked fibres for facial musculature of individual no. 104. Abbreviations: AA: Anterior Auricularis, DAO: Depressor Anguli Oris, DH: Depressor Helicis, DLI: Depressor Labii Inferioris, DS: Depressor Supercilii, LLS: Levator Labii Superioris, LLSAN – Levator Labii Superioris Alaeque Nasi, PA: Posterior Auricularis, SA: Superior Auricularis, SAL: Superior Auriculolabialis. Green muscle labelled as platysma, but see Limitations section—Identification of muscles, for the masseter as an alternative possibility. Similarly, blue muscle labelled as zygomaticus major, but see the Limitations section for some fibres belonging to masseter being a possibility.

**Figure 4.**
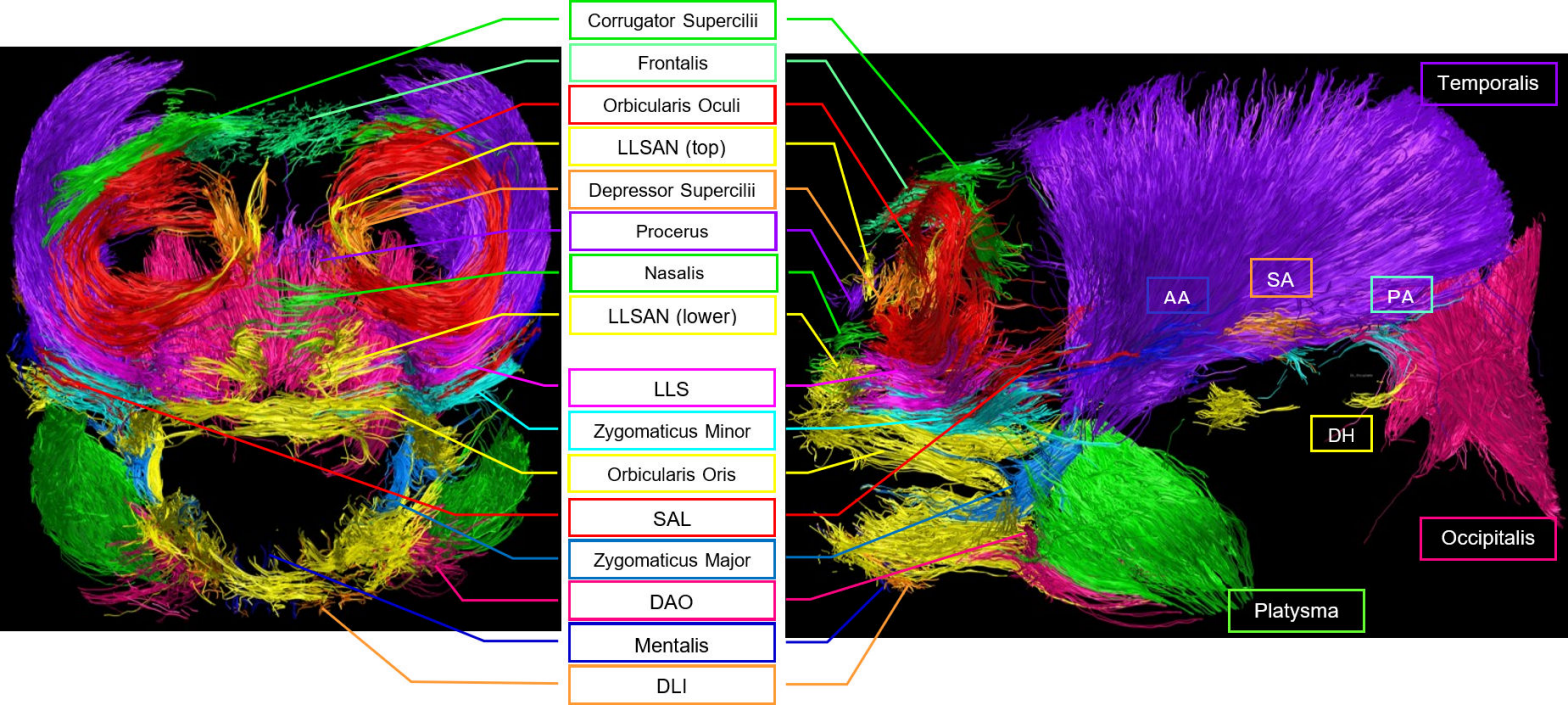
Frontal and lateral view of tracked fibres for facial musculature of individual no. 106. Abbreviations: AA: Anterior Auricularis, DAO: Depressor Anguli Oris, DH: Depressor Helicis, DLI: Depressor Labii Inferioris, DS: Depressor Supercilii, LLS: Levator Labii Superioris, LLSAN : Levator Labii Superioris Alaeque Nasi, PA: Posterior Auricularis, SA: Superior Auricularis, SAL: Superior Auriculolabialis. Green muscle labelled as platysma, but see Limitations section—Identification of muscles, for the masseter as an alternative possibility. Similarly, blue muscle labelled as zygomaticus major, but see the Limitations section for some fibres belonging to masseter being a possibility.

Twenty-two facial muscles were identified with TrackVis (figures 2–4) and then described (§3.1) and analysed (§3.2) in the common marmoset. Video rotations in three-dimensional (pitch and yaw) of the overall facial musculature can be found in electronic supplementary material, videos S2–S7, and videos showing the muscle positions with the MRI image can be found in electronic supplementary material, videos S8–S12.

### 3.1. Anatomical description of tracked muscle fibres

Very few differences were found between the muscles reported in previous dissections and here tracked in terms of origin, insertion and general visualization. In table 2, the anatomical descriptions of each muscle are compiled with any differences found. Differences between the individuals are indicated when found.

**Table 2.** Differences found between the anatomical descriptions of the muscles from previous dissections (table 1), and the tracked muscle fibres here reported (grouped into the respective facial muscles more relevant for the production of AUs in common marmosets [12]), in terms of origin, insertion and general visualization. Differences between the three individuals tracked are also noted.

muscle name	abbreviation	tracking colour	differences from dissections	differences between individuals
Frontalis	F	aqua green	none	none
Corrugator supercilii	CS	green	none	none
Depressor supercilii	DS	orange	none	none
Procerus	Pro	purple	very few fibres found and thus difficult to identify the same clear origin/insertion	difficult to visualize in individual no.102, and In individuals, no. 104 and no. 106, it was depicted as a very small muscle
Orbicularis oculi	OOc	red	none	none
Nasalis	N	green	none	present in all individuals, but only a very small amount was tracked in individual no. 102 and with different fibre direction
Orbicularis oris	OOr	yellow	none	none
Zygomatic major	ZMa	blue	fibres inserted both in the upper and lower portions of the OOr, near the lip corners	none
Zygomatic minor	ZMi	light blue	none	none
Levator labii superioris alaeque nasi	LLSAN	yellow	tracked as a two-portion muscle. The superior portion was a small muscle located between DS and Pro, with one end near F and the contralateral end near N. The inferior portion was tracked as a muscle surrounding the nostrils.	The superior portion was difficult to delineate in individual no. 102.
Levator labii superioris	LLS	pink	origin more lateral to the OOc extending to the zygomatic bone	none
Superior auriculo labialis	SAL	red	none	none
Depressor anguli oris	DAO	dark pink	origin seems to be deeper and more caudally extended into the platysma	none
Depressor labii inferioris	DLI	orange	similar to the DAO, insertion seems to run more caudally along the mandible of the lower lip	none
Mentalis	M	dark blue	none	none
Anterior auricularis	AA	dark blue	none	difficult to track in individual no. 104
Posterior auricularis	PA	light blue	none	difficult to track in individual no. 106
Superior auricularis	SA	orange	fibres origin seemed to be more in the T than the O	not tracked in individual no. 102; direction of fibres seems more superior in individual No. 104 than in no. 106
Depressor helicis	DH	yellow	from the rostral to the ventral side of the auricular base	direction of fibres differed in all individuals
Platysma	Pla	green	none	none
Temporalis	T	purple	none	none
Occipital belly	O	pink	none	in individuals, nos. 102 and 104, it was tracked as a two-layered muscle, with fibres going from the occipital bone or temporal bone to the neck

In addition to the muscles described above, the platysma, temporalis and occipital belly descriptions can be found in the electronic supplementary material, results.

### 3.2. Quantification of tracked muscle fibres

**Figure 5.**
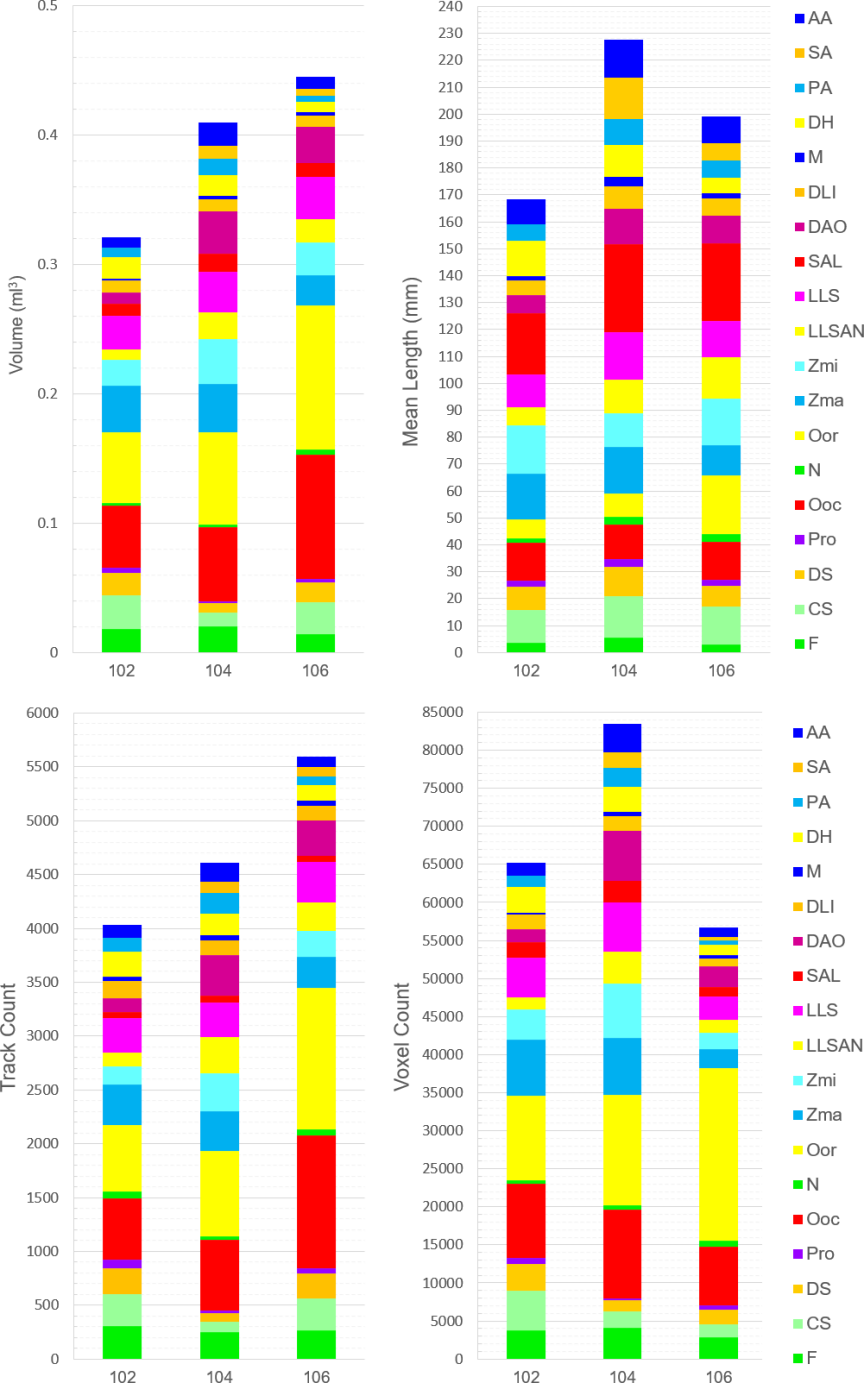
Track count, voxel count, mean length (mm) and volume (ml) of the facial muscles of the three common marmosets (excluding the much larger muscles platysma, occipitalis and temporalis, compared in electronic supplementary material, results). Colours of the muscles in the legend match the colours of the scans (figures 2–4). See table 1 for muscle abbreviations.

Variation in the quantification of tracked muscle fibres was found both between individuals and between the left and right facial muscles in all parameters measured, namely track count, voxel count, mean length and volume, and these parameters were not constant across individuals or muscles (electronic supplementary material, table S2). Overall, individual no. 102 was observed to have lower track count and volume, while individual no. 106 had a higher track count and volume. On the other hand, individual no. 104 had the highest voxel count and the larger mean length. Individual No. 106 had the lowest voxel count, and individual no. 102 had the shortest mean length (figure 5).

As expected by previous dissections of common marmosets, variation of quantification between the muscles was also observed. The largest muscle in all individuals for track count, voxel count and volume was the OOr, closely followed by the OOc. However, the largest muscle by mean length was the SAL in all individuals, with its fibres connecting the ear and mouth, but presenting a small track count, voxel count and volume. Conversely, for track count and mean length, the smaller muscle for individual nos. 102 and 106 was the M, while for individual no. 104, it was the Pro and the N, respectively. For voxel count and volume, individual no. 102 had M as the smallest muscle and individuals nos. 104 and 106 had Pro in both parameters as the smallest muscle (figure 5).

**Figure 6.**
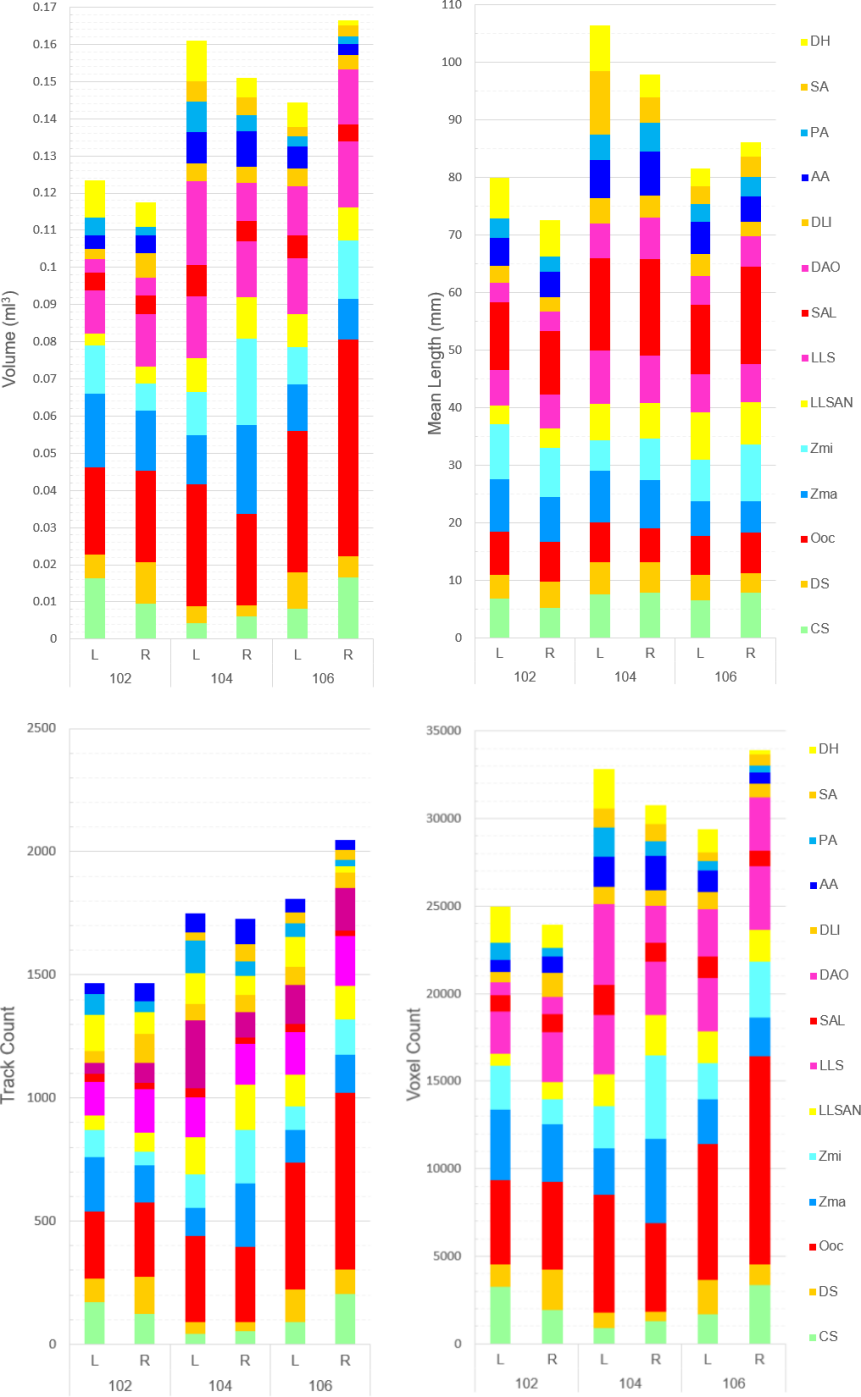
Track count, voxel count, mean length (mm) and volume (ml) of the left and right facial muscles of the common marmoset (excluding the much larger muscles platysma, occipitalis and temporalis, compared in electronic supplementary material). Colours of the muscles in the legend match the colours of the scans (figures 2–4). See table 1 for muscle abbreviations.

For the muscles for which a clear bilateral separation was observed with separate ROI tracking performed (14 out of 22 muscles), quantification of each muscle on the left and right hemifaces was also done (figure 6) to determine possible asymmetries in facial muscles. For track count, overall asymmetry in the facial musculature was only evident in individual no. 106, whose right hemiface had approximately 200 more fibres tracked than the left hemiface, while individuals with nos. 102 and 104 presented similar values. However, both in individuals with nos. 102 and 104, individual muscles presented slight differences, with some muscles having higher track count on one side and others on the other side of the face. These variation patterns also did not seem consistent between the two individuals, as for example, the OOc appeared larger on the right hemiface of individual no. 102 and larger on the left hemiface of individual no. 104. Likewise, for individual no. 106, not all muscles were larger on the right hemiface, despite this side presenting overall higher track count.

For the other parameters, voxel count, volume and mean length, there was also no clear left or right side dominance in asymmetry, but even more differences between hemifaces and muscles were observed. All individuals presented one hemiface more dominant over the other, but in individuals with nos. 102 and 104, the left hemiface presented higher overall values for the three parameters; for individual no. 106, the right hemiface had higher values. As above, there seems to be no clear pattern within hemifaces to which muscles are larger in any of the parameters either.

In addition to the muscles presented above, the platysma, temporalis and occipital belly parameters and comparisons of quantification can be found in the electronic supplementary material, table S2, Results and figure S2.

## 4. Discussion

Facial muscles in common marmosets were successfully visualized and analysed for the first time using 9.4T MRI and DTI. In the current work, 22 facial muscles were successfully tracked, described, quantified and compared in three common marmosets. This work demonstrates for the first time the feasibility and power of this method to accurately track facial muscles in great detail in a species with fairly small facial musculatures. The main goal of this work was to demonstrate the feasibility of the application of MRI-DTI to facial musculature in order to visualize muscles in a digital medium, which has been successfully accomplished in three individuals. As a second goal, we aimed at analysing the visualized muscles through quantification of the fibres tracked, including muscle and individual comparisons. Overall, our work intends to exemplify the use of this method in this particular context and its digital visualization and quantification abilities, rather than being a foundational anatomical study (since this has already been achieved in several past anatomical studies of the common marmoset facial musculature, e.g. [3]) or an empirical study in the stricter sense (since a larger sample would be needed).

We next discuss the potential significance of the results here obtained, followed by a comparison of the performance of this method with previous methods used for facial musculature dissection in this species (reverse dissection and DiceCT). We then present limitations faced currently by this method and opportunities to improve the method and applications in the future directions section.

### 4.1. Description and quantification of the common marmoset facial muscles with MRI-DTI

#### 4.1.1. Muscle presence

The current work found minimal variation in the presence of facial muscles in the common marmosets. All facial muscles found in previous dissections [17,49] were also found here for all individuals, with the exception of the SA, which could not be found in the lightest individual (no. 102).

#### 4.1.2. Facial musculature variation between individuals

The variation found in the overall muscle parameters indicates that the facial musculature varied in terms of its macrostructure between individuals (e.g. number and length of fibres, overall size, etc.), but these parameters do not seem overall to vary proportionally between them, i.e. facial musculature with more overall fibres does not necessarily have overall longer fibres. While overall Track Count and Volume seem to be more or less proportional to the weight of the individuals, voxel count and mean length do not, as the heaviest individual (no. 106) had the lowest voxel count and the mid-weight individual (no. 104) had the highest voxel count and mean length. Hence, in our sample, facial muscles do not seem to vary solely allometrically within the same species. While our sample is too small to extrapolate to other species or perform robust statistical analyses, this information has applications in terms of understanding behavioural variables in a comparative perspective. For example, allometry variation was proposed before between different primate species, in which larger species with larger faces would have a higher number of facial movements (produced by higher number of muscles [50]).

#### 4.1.3. Variation between muscles

The largest muscle in all individuals was the OOr, closely followed by the OOc when compared to the other facial muscles. The mouth produces the highest number of AUs in common marmosets [12] and other primates, including humans (e.g. [51–53]) and so it is not surprising that this is a well-developed robust muscle. As marmosets are a highly communicative species [11,13,54], having a well-developed OOr may also be necessary to shape the mouth in a variety of positions to produce different vocalizations, as well as for facial expression production. It is less clear why the OOc is the second largest muscle, as its function is mainly closing the eye and blinking. Nonetheless, four AUs are produced by this muscle [12], and hence, more than closing the eye, it may be used frequently for communication purposes as well.

Additionally, the common marmoset is an obligate gum-feeder and does so, partially, by creating a large gape to gouge at the tree bark [55,56]. It is possible that this feeding mechanism also influences the size of the OOr, since the lips have to be widely stretched during gum feeding.

In this sample, M, Pro and N muscles seem to be less robust and less developed in the common marmoset. Although the M muscle in most primates, including humans, raises the mental area or chin with AU17—Chin Raiser, in common marmosets, this movement was not reliably identified [12], and hence might not be a muscle often used in this species. Although a nostril movement has been identified in marmosets produced by the N muscle, the AU38—Nostril Dilator [12], this is a very subtle movement, which may explain the need for only a smaller muscle. However, there seems to be some individual variation in this muscle, as only one individual presented shorter mean length for N. Since the Pro muscle has an overlapping function with the DS, and despite these two muscles together producing a very conspicuous and strong movement lowering the glabella in marmosets (AU4—Glabella Lowerer [12]), it seems that the Pro muscle is less relevant in this species. In other species, such as humans, this movement may have differentiated further to create a slightly distinct movement from the one produced by DS, in which the Pro pulls the glabella down and the DS pulls the inner eyebrows down [57].

#### 4.1.4. Asymmetrical variation between hemifaces

No hemiface seemed particularly dominant over the other for track count, both for overall and individual muscles on individuals with nos. 102 and 104. This unilateral absence of a clear dominance pattern was an unexpected result to some extent, given what is known about human facial symmetry; while it is well known that the human face is morphologically and functionally asymmetric [58–60], usually one side of the face is dominant in some aspect for all features, such as the left hemiface being more attractive [61], more expressive [62–64] and producing stronger muscle contractions [65]. Moreover, contradicting the human results, individual no. 106 presented a dominance of the right hemiface for all overall parameters, but as the other individuals, no consistency between side-size bias for each muscle. As very little is known about facial asymmetry in non-human animals, either at an anatomical, morphological or functional level, these data are an important insight into definitely asymmetrical, but not with a clear hemiface bias. Hence, further research is needed to understand why this is the case and to what extent other mammalian species with well-developed facial musculatures and wide facial movement repertoires may or may not present hemifacial asymmetry and what this may mean for the evolution of the facial musculature.

#### 4.1.5. Potential variation origins

Although the marmosets imaged here were bred from the same population, all male and kept in standardized conditions, which would be expected to lower variation in their facial musculature, both for overall and individual muscles, some differences between individuals were found, apparently not due to the weight or skull size. However, the sample was relatively small, and hence the variation found needs to be confirmed with a larger sample of marmosets in future imaging studies. Nonetheless, this variation in overall and individual measures of the common marmoset facial muscles is surprising and may suggest more diversity in facial musculature than previously thought. To better understand this slight variation in facial musculature structure between individuals, we need behavioural data to be collected in future studies and analysed together with the anatomical data.

### 4.2. Comparison of MRI-DTI with reverse dissection and DiceCT

As far as we know, only two techniques have previously been employed to investigate the facial muscles of the common marmoset: manual dissections (traditional: [49] and reverse facial mask: [3]) and DiceCT [17]. With the development of MRI-DTI for facial muscles, the current work is paving the way for an additional powerful technique to visualize and analyse facial muscles. Hence, it is important to discuss how these techniques differ and may contribute to a better understanding of the facial muscles, which are presented in this section.

*Identification of individual muscles:* While the reverse dissection technique conserves more intact musculature than traditional dissections, the DiceCT seems more efficient at picking up on ear musculature due to its small size and other muscles that may be obscured by connective tissue, although most muscles were identified by both techniques [17,30]. With MRI-DTI, all muscles previously identified with reverse dissection and DiceCT were identified, at least in one of the specimens. Ear musculature also proved challenging to track in the current work, as well as the muscles inserting on the upper lip, due to similar direction of fibres and potential branching of the same muscle. Origin and insertion points of muscles are perhaps harder to pinpoint with MRI-DTI and reverse dissection than DiceCT, as blending with other fibres may obscure precise start and end of muscles in reverse dissection; in MRI-DTI, direction changes of tracked fibres at certain points may make it harder to precisely pinpoint this as well. DiceCT seems to be the better method due to the staining process which identifies more accurately these points.

*Direction of fibres:* MRI-DTI visualization and analysis are based on directionality of muscular fibres represented by the voxels, hence it is possible to examine how each muscle fibre is disposed in space, while in the Dice-CT imaging, it is not possible to detect fibre direction; in manual dissections, the detection of this component is usually not visible or it may depend on the preparation and size of the specimen.

*Three dimensions and asymmetry:* Unlike with dissections or DiceCT, in which the facial mask is separated from the skull, MRI-DTI maintains the three-dimensional relative position of the muscles, and hence the three-dimensional information of the musculature is possible extract. This is important to examine, for example, asymmetry in facial musculature, as it has been done before in another digital anatomy method which combines traditional dissection, manual digitization and a three-dimensional modelling protocol [66]. However, it is important to note that modelling of facial muscles is based on heuristic definitions of the muscles that assume homogeneous morphology of each muscle, and only techniques like DiceCT and MRI-DTI can accurately define the inner structure and variation of each muscle [66]. Asymmetry of small muscles such as the facial muscles of the common marmoset may be hard to determine by visual observation only in a manual dissection, and quantification measures might be needed, such as the ones employed in the current work with MRI-DTI. Behaviourally, it is well known that human spontaneous facial expressions are highly asymmetrical [67,68], but it is not so clear if these asymmetries are behavioural or anatomical, as medical texts often assume symmetry in facial muscles, contrasting with evidence from modelling work which found marked asymmetries in left and right muscles [66]. In other species, facial expression asymmetry has not been studied, and hence understanding facial anatomy asymmetry may support future studies of facial expressivity and its meaning in communication and emotion.

*Size of musculature:* Smaller species have smaller facial muscles, and such muscles are harder to examine with a naked eye. Digital anatomy allows the augmentation in size as much as the resolution from digital scans allows. Dice-CT allows, for example, up to 0.05 mm inter-slice sampling, but the contrast between tissues is low and the resolution of the resulting image is also low, while MRI contrast and resolution are both higher and tracking happens in three-dimensional space. On the other hand, simple resolution in MRI is higher, and CT does not distinguish muscle fibres; we can only visualize the stained tissue, which includes nerves, muscles and may include other tissues. The smaller muscle volumes quantified with Dice-CT were 4–10 mm^3^ [30]. While with MRI-DTI, muscle volume cannot be easily and directly quantified but can be estimated based on the voxel volume. In this study, the volume of one voxel was 0.004913 mm^3^ and the volume of the muscles was calculated from the number of voxels that the fibres pathed.

*Versatility of this technique:* In this study, a 9.4T ultra-high-field MRI system was used and the DTI technique was applied, which enabled the description of how detailed muscle fibres run. Although our data are very unique and unprecedented in the study of identifying facial muscles in animals, which used a powerful system, this technique is not restricted to this system only. Certainly, the use of a higher field MRI system is appropriate for identifying more detailed muscle fibre runs in smaller animals, but, for example, Hata and his colleagues used a 7T MRI system and DTI technique to evaluate the process of muscle repairing in mice [69]. For larger animals (e.g. macaques, dogs and cats), a 7T or 3T MRI system is sufficient to describe muscle fibre runs. In addition, lower field MRI can be applied to smaller animals by extending the imaging time. It is expected that the application of this technique (with 9T, 7T or 3T) to a range of animal species will facilitate cross-sectional studies on the evolution of facial muscles and the development of communication through facial expressions.

### 4.3. Limitations of MRI-DTI for identification of facial muscles

Although this method revealed a much more comprehensive description and quantification of facial muscles compared to previous techniques, it still presented some technical challenges and limitations, which are discussed below.

*Sample size:* While demonstrating the feasibility of MRI-DTI application to facial muscles in common marmosets does not require a large sample, larger samples would be preferred for robust quantification of muscles and for broader comparisons between individuals, particularly to perform statistical analysis. Small sample sizes are also common in traditional dissection techniques, both due to availability of samples (e.g. while marmoset heads might be easy to procure due to their availability as a laboratory species, other species, such as gorillas, are not easily available due to their protected status) but also due to all anatomical methods being very time-consuming to apply. In particular, in our work, MRI-DTI applied to facial muscles only allowed us to sample three individuals, although more were available.

*Presence of muscles:* As mentioned before, one muscle could not be found in one individual, which is unclear if this was due to technical issues (e.g. specimen state during fixation, difficulty finding the fibres due to different development of fibres or asymmetries) or if this muscle indeed presents individual variation, which is something that should be considered with this technique.

*Identification of muscles:* The identification of fibres with MRI-DTI was based on previous anatomical knowledge of attachment points and fibre direction of each muscle. However, this method may be challenging to apply when identifying muscles that overlap or intertwine, which may lead to muscle misidentification or inclusion of fibres belonging to different muscles. For example, in individual no. 102, it could be that the muscle identified as the zygomaticus major muscle is part of the buccinator instead, in individuals with nos. 104/106, the platysma may have some mixed fibres from buccinator or masseter, or in individual no. 104, the occipitalis may have mixed fibres from neck musculature. Refinement of these mixed fibres extraction is technically possible through additional ROI placement, accumulating anatomical information and more extensive method application in future studies. This study deals with data from only three marmosets and also discusses the methodology and basic information for such a detailed analysis. More detailed information should be revealed in future studies.

*Minimum size of fibre tracked:* The minimum voxel size analysed here was determined to create less noise, as smaller voxel size creates very noisy data (i.e. grainy images that obscure the tracked fibres). Thus, there might be a limitation in terms of the smaller fibres that can be tracked with MRI-DTI for each species. Nonetheless, if imaging smaller species, voxel sizes can be decreased until images get noise and parameters adjusted until only clear fibrous tissue is seen. Longer fibres are also easier to track with MRI-DTI. Shorter fibres are also possible to track (e.g. ear muscles), but they require better information on where the muscles are located and more time to find and separate the fibres from other longer ones.

*Parameters for fibre tracking:* In this study, the same parameters for tracking fibres were used between individuals; for example, we kept the same ‘skip’ value (i.e. the same number of fibres were skipped in each scan of each individual), so we could compare number of voxels and volumes between individuals in order to quantify and compare the muscle fibres between individuals. However, varying this value in order to achieve a more complete visualization of the facial muscles in each individual revealed more complete facial musculature with more fibre information in some individuals. Hence, because the conditions in which individuals’ facial muscles are fixated may slightly change the resulting conserved tissues, even if the protocol followed is the same, during visualization users need to decide if the priority is the quantification or the visualization of the facial muscles and set the parameters as appropriate. In addition, both the skip factor used and the quality of fibre tracking will directly impact the quantification of the muscle fibres. Hence, numerical validation of the skip factor used should be computed in the future, both at the level of the tracking in the MRI-DTI images (e.g. quantifying muscles and/or individuals with different skip factors) and through comparison with metrics of the same muscle within traditional dissections.

*Difficulty in identifying and discriminating muscle tissue:* Total differentiation of muscle fibres and other tissues is not possible by the software parameters alone; only by manual decisions that are based on the anatomical information is it possible to track solely muscle fibres. One of the decisions on which fibres to track is based on the direction of each fibre, which assumes consistent directionality of muscle bundles. Hence, in previous studies, crossing fibres of the tongue, for example, could not be reported. Multi-tensor models or definitions of more ROIs that allow some flexibility in fibre direction were suggested to solve this issue [39]. In addition, these interdigitated fibres may have a role in stiffening the muscles, which is very relevant for tongue movements, but perhaps less for facial movements, as mimetic muscle function is directed at pulling the skin in different directions more than stiffening. Nonetheless, fibre direction and crossing are important to consider during imaging for facial muscles. In addition, since the fibre tracking is based on manual decisions when placing the ROIs to exclude/include fibres, there might be a degree of bias on the amount of fibres to include, since the tracking is based on previous anatomical knowledge. However, this bias error will only impact the quantification of fibres, but not the presence and attachment points of the muscles.

*User-based tracking decisions and expertise required:* This MRI-DTI method still has some degree of subjectivity due to the ROIs being user-defined and based on the anatomical information available, regarding direction of fibres for each muscle. However, the same criteria and step-by-step method were used in all individuals and muscles, in an effort to reduce subjectivity. In order to further reduce this issue, voxel-based analysis can also be used instead of user-defined ROIs [37]. In addition, this method still requires anatomical expertise as it is difficult to pinpoint origin, insertion and fibre direction without anatomical expertise of the facial muscles. Hence, in the current work, the tracking was verified at several points and discussed between an expert anatomist (AB), an expert in facial movement (CC) and the software expert (KM) in order to achieve a correct tracking of muscle fibres. Hence, this method is not possible to use without expertise or consultation with expert anatomists.

*Costly method:* Although a very promising method for this field, tracking of fibres with ROIs is a manual process that takes a very long time currently and requires some computational power, which increases with the number of ROIs defined. The equipment used here is also a very powerful piece of equipment that is expensive and yet rare, but has the potential to expand and develop due to its specialized applicability. When compared to 7T or lower field scanners, 9.4T scanners have much higher encoding and processing time, and thus a longer period that the individual needs to undergo the scan [70].

*Equipment still in development:* As 9.4T MRI scanners have not been approved for human clinical settings and are only yet used for research, this equipment still needs to go through significant development and testing [71]. Some of the limitations that have been reported include, for example, issues such as image artefacts, increased sensitivity to motion artefacts (including biological functions such as breathing or muscle relaxation [70]) and clinical safety regarding, for example, tissue absorption rate, overheating and optimal coil configuration (although [72] found no health risks in human volunteers). Our study was *ex vivo*, and it is not yet known if there are differences in DTI between *in* and *ex vivo* [73]. However, a brain 9.4T MRI-DTI database scan of *in vivo* common marmosets has been published recently [74], which did not find relevant tracking differences.

*Other parameters standardization:* MRI-DTI results may be affected by the magnetic field intensity, temperature and/or the imaging protocol, and therefore, it is necessary to unify equipment and imaging protocols. It may be difficult to conduct comparative studies between institutions if these parameters cannot be standardized. In this case, direct comparisons of fibre volume and length are not possible.

### 4.4. Conclusions and future directions

Within the rapidly evolving digital anatomy subfield, MRI-DTI is a valuable tool to better understand muscle structure, morphology and function and aid traditional dissections. The high magnetic field 9.4T MRI-DTI used here has not yet been used *in vivo* (due to the time the scans take to produce and current lack of approval for clinical use), it has great potential for tracking facial muscle fibres *in vivo*, for example, with anaesthetized individuals. Lower magnetic field 3T MRI-DTI is already routinely used in several settings, for example, for human leg muscle damage research [75], and higher magnetic field 7T has been used for leg muscle damage in rats [76]. Particularly in smaller species, in which facial muscles are hard to dissect manually, separation of fibres becomes harder the smaller the muscles are, and visualization with a naked eye is almost impossible. The 9.4T MRI-DTI has the ability to generate *in vivo* data with the resolution of 0.1 mm [77], which would allow the imaging of very small muscles in very small species.

If using the same MRI-DTI parameters with a new specimen, the values presented here can be directly compared with results from future scans. This is thus a very promising method, not only that allows detailed analysis of facial musculature of a species, but it opens up the possibility of cross-species comparisons with precise quantification of facial muscles, including other non-human primates, humans and non-primate species, in order to better understand the mechanisms of facial muscle evolution.

Another exciting new application of this method is in the study of individual variation of facial musculature, which has received less attention in the field of anatomy. Traditionally, dissections are performed in a few individuals only and used as representations of whole populations or even species. In the case of humans, research has been discussing that individual differences in facial expressivity exist [78–80], but it is still not clear if these differences stem from anatomical differences or behavioural differences. Hence, this method will also allow better within-species comparison of facial muscle variation between individuals, which may be used not only in research settings to better understand differences in expressivity between people and how this reflects in successful communication, but also, for example, for medical training and applications, ranging from surgery to guided radiation therapy education, particularly if combined with other methods, such as VR analysis [81].

## Data Availability

Our data are uploaded as supplementary material [82].
